# Rat Bite—Followed by Severe Constitutional Symptoms

**Published:** 1872-01-01

**Authors:** Chas. W. Earle

**Affiliations:** 673–5 West Lake Street; Chicago


### RAT BITE—FOLLOWED BY SEVERE CONSTITUTIONAL SYMPTOMS.

BY CHAS. W. EARLE, M.D., CHICAGO.

S. N., æt. 40—Employed by Postoffice Department of this city. This patient is a strong and robust American, and while emptying a box was bitten by a rat in the *abductor pollicis* muscle of the left hand.

The wound healed in two days, and the accident had almost been forgotten, when on July fourth, nine days after the reception of the injury, a small point of inflammation was noticed at the place mentioned above.

I saw the patient the following day and found a hand, hot, swollen and painful. The pulse was full and somewhat accelerated, tongue covered with a whitish coat, and there was evidence of an erysipelatous tendency, as the redness which surrounded the point where the poison was inserted had extended up the forearm to a considerable extent.

I ordered immediately morphia q. s. to allay pain, and a tonic consisting of quinine and iron. As an external application I ordered a linseed poultice upon which a drachm of carbolized glycerine ( ʒ i to ℥ ii) was placed.

The following day the patient was not any better and I increased the dose of quinine and iron.

By the eighth, symptoms began to abate. The ninth and tenth my patient improved. On the eleventh the inflammation had nearly subsided and a hard dark colored core remained. It was the size of a very large hickory nut and perfectly destitute of sensibility.

The iron and quinine was given four times a day and the poultices continued. From the twelfth to the fifteenth of July every thing progressed favorably. My patient experienced very little pain, there was no soreness, and I anticipated a safe and comfortable convalescence for him.

July sixteenth, Mr. N. had occasion to visit one of his rented houses in a distant part of the city. He walked some distance and became quite weary. In fact exerted himself more than he should at this time in his convalescence. The seventeenth he was not as well. There was more heat and swelling in the hand. The day following his hand was still swollen and one of the lymphatics on the anterior aspect of the forearm was inflamed and enlarged. I painted the gland with iodine and increased the dose of quinine and iron.

July nineteenth, I saw him early in the A. M. The gland which I had noticed the day before was not any larger, but three or four above the elbow were now considerably enlarged and very painful, while the lymphatics in the left axilla and one in the right was commencing to trouble him.

He had experienced chilly sensations during the night and was suffering from pain in all parts of his body. His pulse was full and bounding; tongue coated; bowels inclined not to move. Ordered quinine and iron in larger doses every two hours. Moved bowels with Pill Co. Cath. Used iodine freely over enlarged glands. Continued carbolized glycerine.

July twentieth, patient is better. Discontinued tonic, and gave during day 60 grs. of the sulphite and 120 grs. of the Bi-Sulphite of Soda.

July twenty-first, patient is improving, the enlarged glands are disappearing.

July twenty-second, the slough has separated leaving an ulcer 1½ by 1 inch in size to ⅓ inch in depth. Ordered to continue tonic four times a day with a nutritious diet. As an external dressing, I left the following:

℞ Oleum Ricini ℥ ii.

Glycerine ℥ ii.

Carbolic Acid ʒ ii.

Under this treatment the patient rapidly improved and finally resumed his former occupation.

This case to me was interesting in two particulars. (1.) The latent character and time of incubation of the animal poison. (2.) The prompt effect of the tinct. ferri chloride with the quinine. In regard to the time of incubation of this poison, authorities are silent. Holmes has a chapter on animal poisons, but says nothing of the time of incubation. Gross, Ericksen and Druitt are also silent. In this case it was about nine days. The recording of one case proves nothing, but if all the cases of this kind were collected, we might arrive at the correct conclusion.

In conclusion I speak of the treatment. Prof. Andrews has always insisted that tinct. of iron is the remedy *par excellence* for erysipelas. The treatment is not therefore new, but my experience in the case has given me greater confidence in the remedy.

673–5 West Lake Street.

